# Assessment of Sodium Diamine Fluoride (SDF) with Light Curing Technique: A Pilot Study of Antimicrobial Effects

**DOI:** 10.3390/mps5020031

**Published:** 2022-04-07

**Authors:** Jens Wilson, Sarah Swanbeck, Gavin Banning, Tatiana Alhwayek, Victoria Sullivan, Katherine M. Howard, Karl Kingsley

**Affiliations:** 1 Department of Advanced Education in Pediatric Dentistry, School of Dental Medicine, University of Nevada-Las Vegas, 1700 W, Charleston Boulevard, Las Vegas, NV 89106, USA; wilsoj27@unlv.nevada.edu (J.W.); victoria.sullivan@unlv.edu (V.S.); 2 Department of Clinical Sciences, School of Dental Medicine, University of Nevada-Las Vegas, 1700 W, Charleston Boulevard, Las Vegas, NV 89106, USA; swanbs1@unlv.nevada.edu (S.S.); banning@unlv.nevada.edu (G.B.); alhwat1@unlv.nevada.edu (T.A.); 3 Department of Biomedical Sciences, School of Dental Medicine, University of Nevada-Las Vegas, 1001 Shadow Lane Boulevard, Las Vegas, NV 89106, USA; katherine.howard@unlv.edu

**Keywords:** sodium diamine fluoride (SDF), light curing technique, antimicrobial

## Abstract

Silver diamine fluoride (SDF) has been useful in clinical dentistry for the purpose of caries arrest and prevention. Although methods for the application of SDF are well-known among dental professionals, such as microbrush applications, few studies have explored the effect of light curing, which accelerates precipitation onto dentin, and whether this has any effect on the antimicrobial properties of SDF. To assess this technique, single (*Streptococcus gordonii*) and polymicrobial (mixed salivary) colonies were grown and plated using SDF applied to hydroxyapatite discs with and without treatment with curing light. Kirby–Bauer Zone of Inhibition assay results revealed no significant differences in the areas between the two treatment groups (SDF: 1.27 mm, SDF plus curing light: 1.25 mm), *p* = 0.887 in the single culture (*S. gordonii*) experiments. In addition, no significant differences were found between the two treatment groups (SDF: 1.26 mm, SDF plus curing light: 1.24 mm), *p* = 0.771 in the polymicrobial culture experiments. Although there may be specific properties associated with SDF induced following light curing, these differences do not appear to be associated with the antimicrobial properties affecting gram-positive or polymicrobial films.

## 1. Introduction

Silver diamine fluoride (SDF) has been useful in clinical dentistry for the purpose of caries arrest and prevention [1,2]. The use of SDF and atraumatic restorative treatment or ART (the use of sealants to prevent caries and restorations to repair lesions) have been tremendously successful in arresting active caries, particularly in primary dentition [3,4]. Recent attention focused on SDF therapy due to the rapid and inexpensive nature of this formulation to quickly eliminate most cariogenic bacteria and promote remineralization, which has established useful parameters regarding the minimum inhibitory concentrations (MIC) and effective inhibitory concentrations (IC50) [5,6].

The mechanism of action for SDF formulations is reliant upon two major components with distinct mechanisms of action, silver and fluoride [7]. First, positively charged silver ions (Ag+) are well-known antimicrobial agents that function to disrupt microbial membranes, create membrane permeability, produce reactive oxygen species and disrupt microbial DNA replication [8,9]. Next, fluoride works with salivary calcium and phosphate to remineralize and harden teeth against pathogenic bacteria and their metabolites [10,11]. Fluoride also functions as a potent antimicrobial due to its intrinsic ability to block the glycolysis-pathway-specific enzyme of this saccharolytic bacteria: enolase [12,13].

Methods for the application of SDF are well-known among dental professionals, and mainly involve the standard microbrush application technique [14,15]. A few recent studies have explored the potential effects of a new application technique—SDF with light curing, both in vitro and ex vivo—which may accelerate precipitation of silver ions onto dentin and significantly alter penetration depth [16,17]. However, neither study conducted an evaluation of whether this technique has any effect on the antimicrobial properties of SDF.

Based upon the lack of evidence regarding this newer technique (SDF application combined with light curing) for this increasingly common dental practice, the overall objective of this project was to explore the antimicrobial properties of SDF on oral microbes with and without light curing.

## 2. Methods

### 2.1. Bacterial Strains

Bacterial cultures were obtained from the American Type Culture Collection (ATCC; Manassas, VA). *Streptococcus gordonii* #35105, *Porphyromonas gingivalis* #33277 and Mixed Bacteria #55644 (containing a mixture of aerobic BSL-1 bacteria characterized as: *Aeromonas* sp. (ATCC 55641; DAP 119 and ATCC 55642; DAP 68), *Corynebacterium* sp. (ATCC 55643; DAP 66), *Pseudomonas* sp. (ATCC 55645; DAP 111, ATCC 55646; DAP 70, ATCC 55647; DAP 631, ATCC 55648; DAP 622), and *Zoogloea* sp. (ATCC 55649; DAP 73) were thawed and placed into Luria-Bertani (LB) broth consisting of 10 g of tryptone (DFO 123-08-04), 10 g of sodium chloride (7647–14-15) and 5 g of yeast extract (BP1422-100), all from Fisher Scientific (Fair Lawn, NJ, USA), and dissolved into 1000 mL of distilled water (autoclaved prior to use in the liquid cycle). Overnight, 250 mL of broth was inoculated and cultured in a bacterial culture with rotary shaking at 90 RPM at 37 °C.

### 2.2. Bacterial Plating

Spread or “lawn” plates were prepared by placing 0.5 mL of bacterial liquid culture diluted to an optical density (OD) of 0.8 onto LB agar plates consisting of LB broth with the addition of 15 g of agar. L-shaped bacterial cell spreaders (14-665-230; Fisher Scientific, Fair Lawn, NJ, USA) were then used to spread the heavy, dense bacterial cultures evenly over the surface of the LB growth media plates. Plates were allowed to incubate overnight at 37 °C upside down in a bacterial incubator. This procedure is commonly used to prepare bacterial plates for assessing antibiotic resistance using Zone of Inhibition tests or ZIT.

### 2.3. Zone of Inhibition Test for Antimicrobial Activity (ZIT-AA)

Hydroxyapatite (HA) disc coupons (0.5 inch diameter; NC1601276) were obtained from Fisher Scientific (Fair Lawn, NJ, USA). For each bacterial plate, approximately 100 μL of sodium diamine fluoride (SDF) was applied to four HA discs using the microbrush application technique. Two of the discs were plated directly onto the bacterial plate without light curing (negative control) and the remaining two discs were then subjected to LED light curing (experimental group) using the Kerr Demi Plus from Kerr Dental (Plymouth, MA, USA) for 20 s to replicate the protocols used in previous studies (and the standard amount of time allotted on this device per run), as outlined in previous studies of SDF with light curing [16,17]. Two plates were processed during each trial run (duplicates) and the experiment was repeated three times (replicates) for a total sample size of n = 12 in both the control and experimental groups.

This dental light curing device produces a narrow spectrum of blue light in the 400–500 nm range, with a peak wavelength of approximately 460 nm. The two light-cured discs were plated SDF-side-down on the bacterial lawn on the experiment-labeled side of the test plate with the two control (non-cured) discs plated on the control-labeled side of the test plate. The zone of inhibition was measured at 24 h using a measuring scale and was recorded and digitized using a Canon PowerShot camera.

### 2.4. Statistical Analysis

Descriptive statistics from the ZIT-AA were obtained from the raw measurements, carried out in triplicate by two different observers. Differences between the measurements were analyzed using two-tailed Student’s *t*-tests in Microsoft Excel, which was appropriate software for parametric data analysis and comparison.

## 3. Results

**Figure 1:**
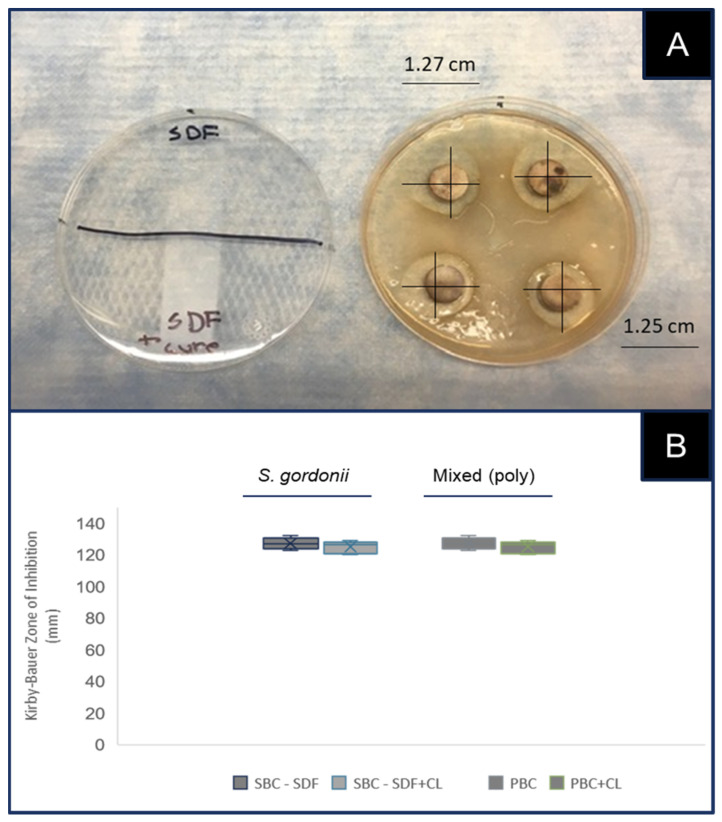
Zone of Inhibition test for antimicrobial activity (ZIT-AA) with Gram-positive and mixed bacterial cultures. (**A**) Visual inspection of ZIT-AA revealed that all treatments (SDF, SDF with light curing) created distinct ZIT-AA. (**B**) Measurements for ZIT-AA and single bacterial culture or SBC (*S. gordonii*) for SDF = 127.16 mm, and 125.2 mm with SDF combined with light curing: *p* = 0.887. The ZIT-AA for the polybacterial culture or PBC (mixed bacteria) with SDF = 126.11 mm, and 124.5 mm with SDF combined with light curing, *p* = 0.771. Graph displays box-and-whisker plot, which displays upper and lower quartiles, with mean denoted by X.

To evaluate the effectiveness of SDF with and without curing light, single bacterial Gram-positive cultures (SBC) and mixed or polybacterial cultures (PBC) were plated for the Kirby–Bauer Zone of Inhibition test for antimicrobial activity or ZIT-AA (Figure 1). The visual inspection of each plate revealed a distinct zone of inhibition immediately surrounding the HA disc coupons treated with SDF, both with and without light curing (Figure 1A). Several (n = 4) measurements of the zone diameter were taken around each disc to determine the average of each set. Three replicates of each experiment were performed, averaged and graphed (Figure 1B).

More specifically, these data revealed that the ZIT-AA for the Gram-positive SBC (*S. gordonii*) was 127.16 mm with SDF and 125.2 mm with SDF combined with light curing, which was not statistically significant, *p* = 0.887. Similarly, the ZIT-AA for the PBC (mixed bacteria) was 126.11 mm with SDF and 124.5 mm with SDF combined with light curing, which was also not statistically significant, *p* = 0.771.

Data for these ZIT-AA measurements were compiled and analyzed to determine if any large variations were observed between the experimental replicates (Table 1). These data demonstrated that the averages for SBC and PBC with SDF under each experimental treatment (alone or with curing light) were very similar at 124.5 mm to 128.2 mm with standard deviations ranging between 3.43 mm and 3.62 mm. The measurements of ZIT-AA between all of the experimental treatments revealed ranges that exhibited less than a 10% variation from the lowest measurement of 120 mm to the highest measurement of 132 mm.

**Figure 2:**
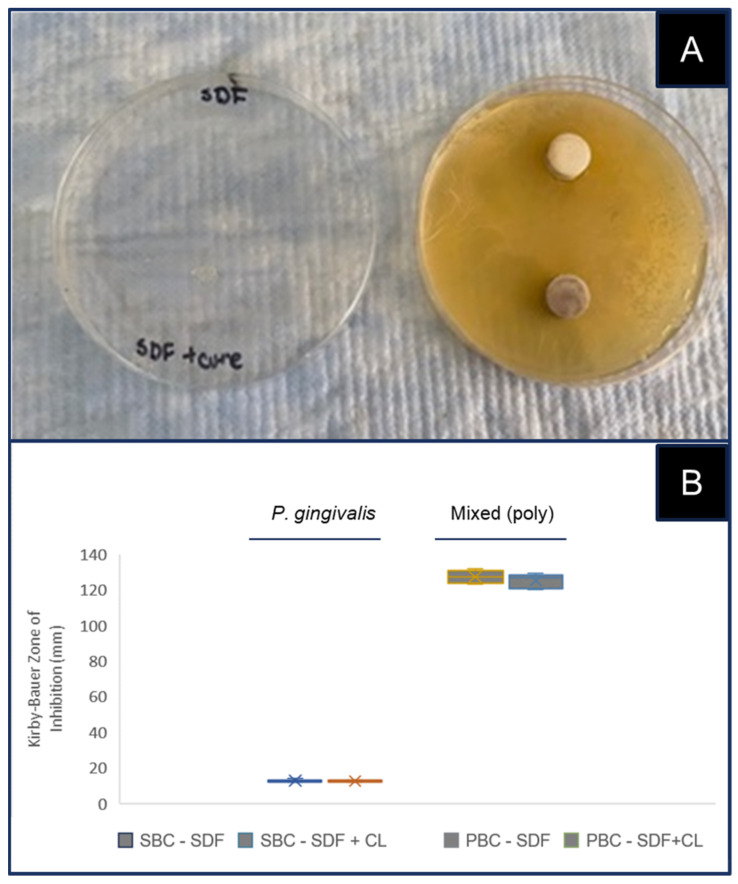
Zone of Inhibition test for antimicrobial activity (ZIT-AA) with Gram-negative and mixed bacterial cultures. (**A**) Visual inspection of ZIT-AA revealed no ZIT-AA with the Gram-negative culture (SDF, SDF with light curing). (**B**) Measurements for ZIT-AA and single bacterial culture or SBC (*P. gingivalis*) for SDF = 12.6 mm, and 12.5 mm with SDF combined with light curing: *p* = 0.6879. The ZIT-AA for the polybacterial culture or PBC (mixed bacteria) with SDF = 126.4 mm, and 125.8 mm with SDF combined with light curing: *p* = 0.818. Graph displays box-and-whisker plot, which displays upper and lower quartiles, with mean denoted by X.

To evaluate the effectiveness of SDF with and without curing light, a negative control using a Gram-negative asaccharolytic (*P. gingivalis*) bacterial culture (SBC) and mixed or polybacterial cultures (PBC) was plated for the Kirby–Bauer Zone of Inhibition test for antimicrobial activity or ZIT-AA (Figure 2). The visual inspection of each plate revealed an extremely limited and barely visible Zone of Inhibition immediately surrounding the HA disc coupons treated with SDF, both with and without light curing and this negative control SBC (Figure 2A). Several (n = 4) measurements of the zone diameter were taken around each disc to determine the average of each set and three replicates of each experiment were performed, averaged and graphed (Figure 2B).

More specifically, these data revealed the ZIT-AA for the Gram-negative SBC (*P. gingivalis*) was 12.6 mm with SDF and 12.5 mm with SDF combined with light curing, which was not statistically significant: *p* = 0.6879. As previously observed, the ZIT-AA for the PBC (mixed bacteria) was 126.4 mm with SDF and 125.8 mm with SDF combined with light curing, which was also not statistically significant: *p* = 0.818.

Data for these ZIT-AA measurements were compiled and analyzed to determine if any large variations were observed between the experimental replicates (Table 2). These data demonstrated that the averages for SBC with SDF for the Gram-negative bacterial culture (*P. gingivalis*) either alone or with curing light were very similar: 12.6 mm to 12.5 mm with standard deviations ranging between 0.81 mm and 0.55 mm. The measurements for the PBC were similar to the first set of experiments with averages of 126.4 and 125.8 with SDF alone or with curing light, with standard deviations ranging from 2.99 mm to 3.12 mm.

## 4. Discussion

Many studies have proven the effectiveness of SDF to arrest caries and limit caries progression in primary teeth, while new methods for the application of SDF with curing light have gained significant attention due to their potential to alter penetration depth [16,17]. In addition, these recent studies have also shown that this method may have the potential to influence the surface hardness of dentin surrounding and below the treated caries lesion [18,19]. However, to date, no study has conducted an evaluation of whether this technique has any effect on the antimicrobial properties of SDF.

Based upon this lack of information, this study successfully explored the antimicrobial properties of SDF on oral microbes with and without light curing and found that, although SDF does inhibit the growth of Gram-positive saccharolytic bacteria and polymicrobial (mixed) cultures, the effect of light curing does not appear to significantly alter the effectiveness of this biomaterial [20,21]. Although the antimicrobial properties of SDF are well-documented and provide substantial evidence for the effectiveness of this treatment [22,23], the application of light curing does not appear to significantly alter these properties—as assessed by the ZIT-AA assay in this study. Even though these results do not demonstrate that light curing increases or potentiates antimicrobial properties, the fact that light curing does not reduce or inhibit this function may be a significant finding given that light curing was previously demonstrated to increase the depth of penetration into dentin and may also help to facilitate dentin hardness. Based upon these combined observations, it appears that light curing does not significantly reduce or alter the antimicrobial properties of SDF in vitro (current study), which would be a counterproductive outcome, but may instead produce other positive outcomes, such as the penetration depth of dentin in vivo (previous studies) and would, therefore, represent a net positive outcome effect.

Given the recent increased attention to SDF in caries prevention efforts and dental education curricula, an examination of the potential effects due to alterations in the application of SDF, such as light curing, are warranted [24,25,26]. This may be the first study to explore and evaluate any potential change in antimicrobial properties of SDF with light curing and allows for a more complete understanding of whether this technique improves or alters antimicrobial properties. As more and more pediatric dentists are changing their attitudes toward the expansion of SDF use, with populations involving behaviorally challenged, anxious and medically fragile children, understanding the implications of new application techniques and approaches becomes more important [27,28].

Despite the significance of these findings, this study also has limitations that should be considered. For example, this was a pilot study conducted in the laboratory (in vitro) and may not represent the exact responses of these bacteria in the complex environment of the oral cavity (in vivo). In addition, due to time constraints and financial limitations, only a limited number of oral bacteria could be evaluated, which may not represent the full spectrum of oral organisms present in any oral biofilm or community [29,30]. Although some preliminary data were analyzed, future studies may include a more comprehensive range of organisms and evaluate the effectiveness of SDF with and without light curing in clinical settings to more accurately assess any potential changes to antimicrobial properties [31]. In addition, direct comparisons between light curing devices and alternative light curing cycles might further elucidate the potential protocols and parameters that influence the effectiveness of SDF with light curing [32,33].

## 5. Conclusions

Although recent studies have demonstrated that SDF in conjunction with curing lights may increase silver ion precipitation and dentin hardness ex vivo and in vitro, the results of this study evaluated the antimicrobial properties of SDF with and without curing light and found no significant differences between these treatments. Although there may be specific properties associated with SDF induced by light curing, these differences do not appear to be associated with the antimicrobial properties that affect Gram-positive or polymicrobial films.

## Figures and Tables

**Table 1:** ZIT-AA measurements for the single Gram-positive (SBC) and polybacterial cultures (PBC).

	SBC (*S. gordonii*) SDF Only	SBC (*S. gordonii*) SDF, Curing Light	PBC (Mixed) SDF Only	PBC (Mixed) SDF, Curing Light
Average STD	127.2 mm +/−3.43 mm	125.2 mm +/−3.86 mm	126.1 mm +/−3.51 mm	124.5 mm +/−3.62 mm
Range	123–132 mm	121–129 mm	122–130 mm	120–128 mm

**Table 2:** ZIT-AA measurements for the single Gram-negative (SBC) and polybacterial cultures (PBC).

	SBC (*S. gordonii*) SDF Only	SBC (*S. gordonii*) SDF, Curing Light	PBC (Mixed) SDF Only	PBC (Mixed) SDF, Curing Light
Average STD	12.6 mm +/−0.81 mm	12.5 mm +/−0.55 mm	126.4 mm +/−3.12 mm	125.8 mm +/−2.99 mm
Range	12–15 mm	12–14 mm	122–130 mm	120–128 mm

## Data Availability

The data presented in this study are available on request from the corresponding author.
